# Multiclass classification of breast cancer histopathology images using multilevel features of deep convolutional neural network

**DOI:** 10.1038/s41598-022-19278-2

**Published:** 2022-09-16

**Authors:** Zabit Hameed, Begonya Garcia-Zapirain, José Javier Aguirre, Mario Arturo Isaza-Ruget

**Affiliations:** 1 eVida Research Group, University of Deusto, Bilbao, 48007 Spain; 2 Bioaraba Health Research Institute, Oncology Diagnostics and Therapeutics Area, Department of Pathological Anatomy, University Hospital of Alava, Vitoria, 01009 Spain; 3 NanoBioCel Research Group, School of Pharmacy, University of the Basque Country (UPV/EHU), Vitoria, 01006 Spain; 4 Biokeralty Reseach Institute, Vitoria, 01510 Spain; 5 Fundación Universitaria Sanitas, Bogotá, 110131 Colombia

**Keywords:** Biomedical engineering, Computer science, Image processing, Machine learning

## Abstract

Breast cancer is a common malignancy and a leading cause of cancer-related deaths in women worldwide. Its early diagnosis can significantly reduce the morbidity and mortality rates in women. To this end, histopathological diagnosis is usually followed as the gold standard approach. However, this process is tedious, labor-intensive, and may be subject to inter-reader variability. Accordingly, an automatic diagnostic system can assist to improve the quality of diagnosis. This paper presents a deep learning approach to automatically classify hematoxylin-eosin-stained breast cancer microscopy images into normal tissue, benign lesion, in situ carcinoma, and invasive carcinoma using our collected dataset. Our proposed model exploited six intermediate layers of the Xception (Extreme Inception) network to retrieve robust and abstract features from input images. First, we optimized the proposed model on the original (unnormalized) dataset using 5-fold cross-validation. Then, we investigated its performance on four normalized datasets resulting from Reinhard, Ruifrok, Macenko, and Vahadane stain normalization. For original images, our proposed framework yielded an accuracy of 98% along with a kappa score of 0.969. Also, it achieved an average AUC-ROC score of 0.998 as well as a mean AUC-PR value of 0.995. Specifically, for in situ carcinoma and invasive carcinoma, it offered sensitivity of 96% and 99%, respectively. For normalized images, the proposed architecture performed better for Makenko normalization compared to the other three techniques. In this case, the proposed model achieved an accuracy of 97.79% together with a kappa score of 0.965. Also, it attained an average AUC-ROC score of 0.997 and a mean AUC-PR value of 0.991. Especially, for in situ carcinoma and invasive carcinoma, it offered sensitivity of 96% and 99%, respectively. These results demonstrate that our proposed model outperformed the baseline AlexNet as well as state-of-the-art VGG16, VGG19, Inception-v3, and Xception models with their default settings. Furthermore, it can be inferred that although stain normalization techniques offered competitive performance, they could not surpass the results of the original dataset.

## Introduction

According to Global Cancer Statistics 2020, breast cancer is the most common malignancy and the primary cause of cancer-related mortalities in the female population worldwide^1^. Specifically, 2.26 million (11.7% of the total cancer incidence) women were diagnosed, with a mortality of 0.69 million (6.9% of the total cancer deaths) during 2020^1^. Therefore, the premature understanding of breast tumor pathophysiology is crucial, which may help in reducing the morbidity and mortality rates in women worldwide. This malignancy is considered a heterogeneous collection of diseases with distinct biological, clinical, and treatment response behaviors^2^. It mainly occurs due to abnormalities in the epithelial tissues of the breast and may invade the adjacent stroma, mammary duct, or lobes^3^. The routine clinical analysis of breast cancer can be carried out by exploiting numerous radiology images, including ultrasound, mammography, and Magnetic Resonance Imaging (MRI)^4,5^. Nevertheless, these non-invasive methodologies might not characterize the heterogeneous behaviors of breast tumors effectively. Therefore, the pathological study is followed as a benchmark to comprehend the pathophysiology of breast tumors. In this method, tissue samples are collected and mounted on glass slides, and subsequently stained these slides for a better portrayal of tumoral morphological and inmunophenotypical characteristics^6^. After that, pathologists proceed with the microscopic examination of these slides to conclude a possible diagnosis of breast cancer^6^. The complete steps of the histopathological procedure have been discussed in^7^ and^8^.

However, the manual interpretation of histopathology images can be a tedious and time-consuming process, and may lead to biased results. Moreover, the morphological criteria used during the manual analysis depend on the domain experience of the pathologists involved. For instance, one study revealed that the overall concordance rate of diagnostic interpretation among participating pathologists was around 75%^9^. To that end, the computer-aided diagnosis (CAD)^4,6,10^ can help pathologists to improve diagnostic accuracy by reducing inter-pathologist variations during the diagnostic process of breast cancer. Nonetheless, traditional computerized diagnostic approaches, ranging from rule-based systems to machine learning methods, may not be sufficient to deal with the inter-class consistency and intra-class variability of complex-natured histopathology images of breast cancer. Furthermore, these conventional methodologies usually leverages feature extraction techniques such as scale-invariant feature transform^11^, speed robust features^12^ and local binary patterns^13^, all of which are dependent on supervised information and hence may cause biased results when classifying these images. Therefore, the demand for an efficient and effective diagnosis yielded an advanced set of computational models based on numerous layers of nonlinear processing units, known as deep learning^14,15^.

In vision-related tasks, the convolutional neural network (CNN)^16^ is considered superior to traditional multilayer perceptron for having translational equivariance and translational invariance properties, the former resulting from parameter sharing and the latter from pooling operations^14,15^. Especially, deep CNN architectures have made significant progress over the last decade among which AlexNet^17^ is considered as the earliest deep CNN model to achieve decent accuracy on the ImageNet Large Scale Visual Recognition Challenge (ILSVRC) during 2012. Subsequently, VGG network^18^ was presented with a novel idea of utilizing a deep network with small-sized convolutional filters, and it secured second position at the ILSVRC during 2014. At this point, Szegedy et al.^19^ introduced the Inception architecture by staking multiple smaller convolutional filters to obtain an effective receptive field, and attained first place at the ILSVRC in 2014. The following year, He at al.^20^ pointed out that increasing the network depth after certain level may degrade its performance and they employed residual connections to overcome this problem, and earned first position at the ILSVRC in 2015. Consequently, numerous state-of-the-art studies leveraged the aforementioned architectures, pre-trained on ImageNet, to accurately classify breast cancer histopathology images using publicly available datasets, including BreakHis^21^ and BACH^22^ datasets. For instance, Jiang et al.^23^ proposed a modified ResNet model^20^ and achieved state-of-the-art accuracy for multiclass classification on BreaKHis dataset^21^. Similarly, the top studies of the BACH challenge^22^ exploited either a single pre-trained network or an ensemble of pre-trained architectures for multiclass classification of breast microscopy images. Recently, Elmannai et al.^24^ acknowledged the effectiveness of Inception modules and residual connections as feature extractors, and achieved state-of-the-art performance on the BACH dataset^22^. To this end, we leveraged the Xception model^25^, stands for extreme inception, which is based on the efficient utilization of Inception and residual connections (see “Proposed model” section). As a feature extractor, it can provide consistent results in the classification of histopathology images of different magnification levels^26^. Our approach effectively utilizes the concepts introduced in^25–29^ to extract salient features from histopathology images using the pre-trained Xception model^25^ as a feature extractor.

The rationale and significance of this study are as follows: 1) To annotate and prepare a private dataset aimed to classify breast cancer histopathology images into normal tissue, benign lesion, in situ carcinoma, and invasive carcinoma^22^. It should be noted that the dataset prepared in this study is an extension of our previously published work on binary classification^8^. 2) To evaluate the performance of four widely used stain normalization methods^28^. 3) To propose a deep learning model based on multilevel features extracted from intermediate layers of the pre-trained Xception model^25^. 4) To optimize the proposed model for the accurate classification of breast cancer histopathology images on the original and normalized images, especially for carcinoma classes. To our knowledge, this is the first study that annotated a new private dataset, proposed a generalized as well as a computationally efficient model based on the Xception network^25^ as a feature extractor, and evaluated the results of four widely used stain normalization approaches^28^. In summary, our proposed model provided consistent results for the definite classification of breast cancer histopathology images into four classes and also outperformed state-of-the-art results.

The remaining sections of this paper are organized as follows. “Methods” section describes materials and methods along with the proposed model. “Results” section explains the findings, and “Discussion” section compares the results of our proposed framework to state-of-the-art research. Finally, “Conclusion” section summarizes the conclusion as well as the future prospects of this work.

## Methods

In this section, we presented the dataset used in this study, followed by the analysis of four stain normalization techniques. Then, we elucidated the training criteria and in-place data augmentation used in this work. Next, we explained the proposed model and its implementation setup. Lastly, we described the model evaluation and the hyperparameter optimization of our proposed model.

### Colsanitas dataset

**Figure 1:**
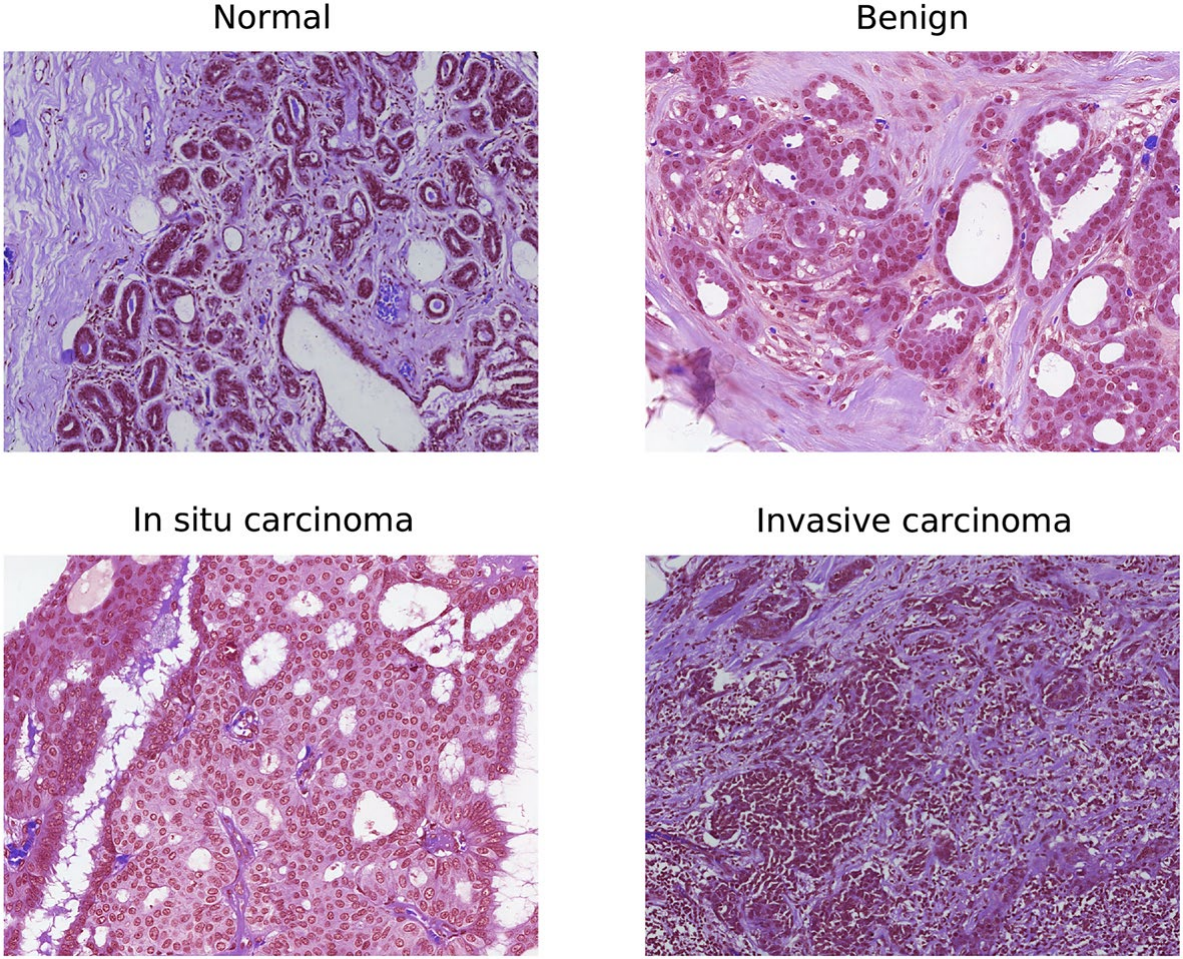
An example of H &E stained normal tissue, benign lesion, in situ carcinoma, and invasive carcinoma from our collected dataset.

In this study, we used the same dataset as presented in^8^ which contains 544 whole slide images (WSIs), retrieved from 80 breast cancer patients at the pathology department of Colsanitas clinic with a dependence of the Sanitas University, Bogotá, Colombia. The protocols followed to convert histology samples into their corresponding digital images are discussed in^8^, including collection and fixation, dehydration and clearing, paraffin embedding, staining and mounting, and digitalization^30^. The tissues were scanned at high magnification (40) using a Roche iScan HT scanner (https://diagnostics.roche.com/global/en/products/instruments/ventana-iscan-ht.html). The WSI images are stained with hematoxylin and eosin (H &E) and illustrate multiple cases from each patient, as explained in^8^. It is worthy to mention that the dataset annotated for our previously published work^8^ contained merely 845 images aimed at binary classification. Whereas the dataset annotated for the current study includes 2250 images formulated for multiclass classification^22^. Two experienced pathologists examined the H &E-stained WSI images and extracted 2250 images, including 600 normal tissues, 250 benign lesions, 250 in situ carcinoma, and 1150 invasive carcinoma. These images were exported as original pixels in .tiff format using Qupath 0.2.3 software^31^. The dimensions of these images are same as that of the BACH dataset^22^ ($2048 \times 1536$ pixels), with a pixel size of $0.46\,\upmu \mathrm{m} \times 0.46\,\upmu \mathrm{m}$. The complete characteristics of our created dataset is provided in Table 1. Also, the examples of normal tissue, benign lesion, in situ carcinoma, and invasive carcinoma images from the Colsanitas dataset are illustrated in Fig. 1.

**Table 1:** Characteristics of our collected Colsanitas dataset.

Image	Quantity	Size ($w \times h \times c$)	Pixel size	Colour	Staining
Normal	600	$2048 \times 1536 \times 3$	$0.46\,\upmu \hbox {m} \times 0.46\,\upmu \hbox {m}$	RGB	H &E
Benign	250	$2048 \times 1536 \times 3$	$0.46\,\upmu \hbox {m} \times 0.46\,\upmu \hbox {m}$	RGB	H &E
In situ	250	$2048 \times 1536 \times 3$	$0.46\,\upmu \hbox {m} \times 0.46\,\upmu \hbox {m}$	RGB	H &E
Invasive	1150	$2048 \times 1536 \times 3$	$0.46\,\upmu \hbox {m} \times 0.46\,\upmu \hbox {m}$	RGB	H &E
Total	2250	$2048 \times 1536 \times 3$	$0.46\,\upmu \hbox {m} \times 0.46\,\upmu \hbox {m}$	RGB	H &E

### Preprocessing

**Figure 2:**
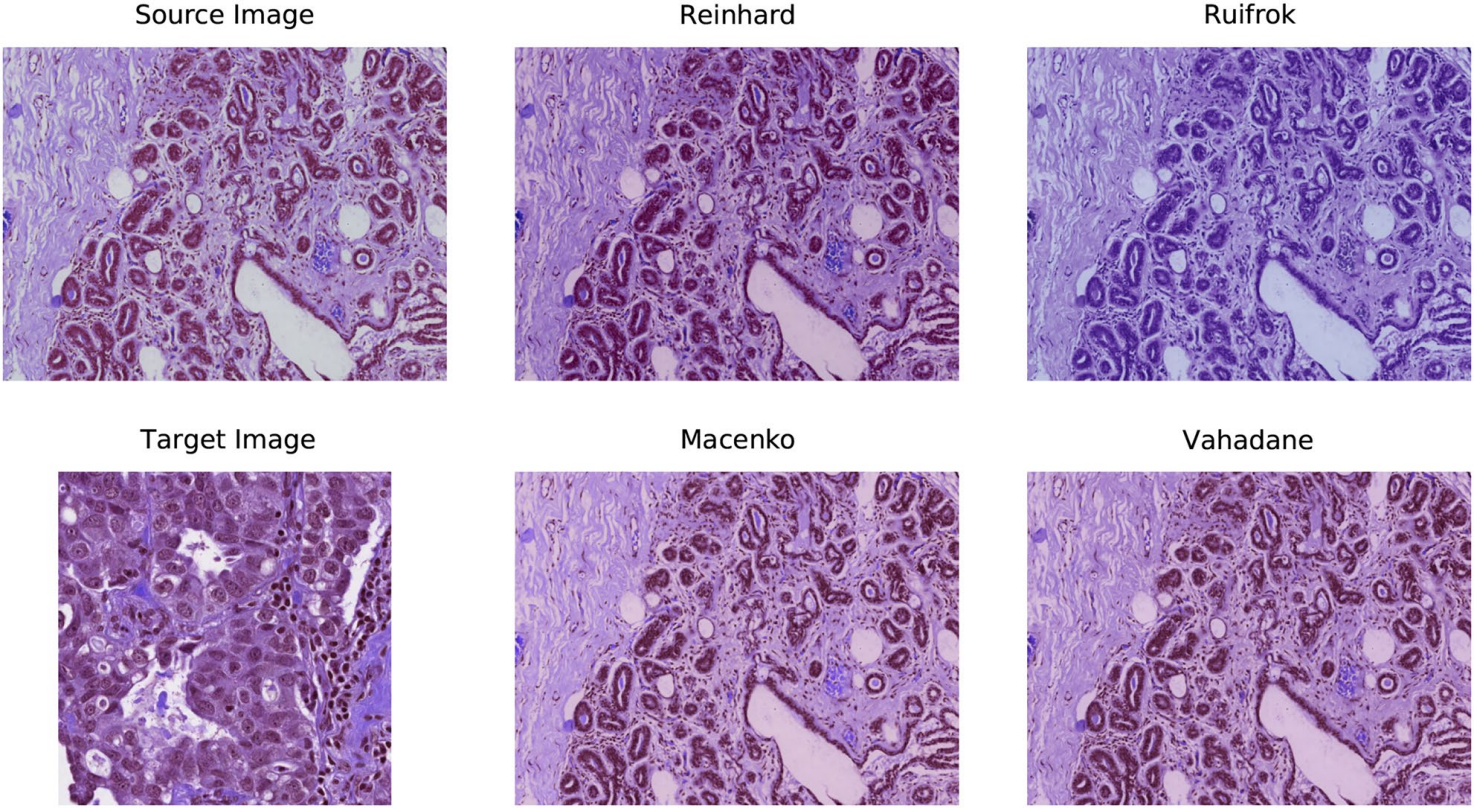
An example of H &E-stained source image, target image, and four preprocessed images resulting from Reinhard^32^, Ruifrok^33^, Macenko^34^, and Vahadane^35^ stain normalization.

The datasets used in this work contain breast cancer histopathology images retrieved from H &E-stained whole-slide images. However, the stain concentration cannot be maintained in all the slides which may result in contrast differences among the exported images. These colour variations in acquired images may affect the performance of computer-aided diagnostic systems^30^. Lyon et al.^36^ highlighted the need for the normalization of reagents and procedures in histopathological practice. Therefore, various colour preprocessing techniques, including colour-transfer and colour-decovolution, are introduced in the literature to standardize the stain appearance. For instance, Reinhard et al.^32^ developed a colour-transfer methodology in which RGB-format images are mapped to the colour distribution of a target image. In this method, a natural RGB image is first converted to a perceptual colour space with decorrelated axes, called $l\alpha \beta$. The mean values and standard deviations of each channel are then adjusted in both the images (source and target) in the colour space. Finally, the $l\alpha \beta$ colour space is converted to get a normalized RGB image. However, this type of global normalization is based on the unimodal distribution of pixels in each channel of colour space, which may not be appropriate when using multiple coloured stains. Therefore, numerous studies have concluded that stain separation prior to stain normalization has a relatively significant impact on the experimental results. For instance, Ruifrok et al.^33^ introduced a colour-deconvolution method to separate the stains. For each stain in a histopathology image, the individual RGB channels are first transformed to their respective optical density (OD) values using Lambert-Beer’s law. Then, the orthogonal transformation of OD values is carried out to get independent information regarding individual stains. Next, the OD vectors are normalized to achieve an unbiased absorption factor for each stain. After that, the normalized OD vectors are combined to form a normalized OD matrix. Lastly, a normalized image is created by leveraging the normalized OD matrix. In the following years, Macenko et al.^34^ also followed a colour-deconvolution approach and concluded that H &E stains can be separated linearly in an OD colour space. First, a histology image is converted to its OD values using the logarithmic transformation. Then, singular value decomposition (SVD) is applied to OD tuples to obtain a two-dimensional plane corresponding to the two largest singular values. Next, these OD-transformed pixels are projected onto the plan and normalized to unit length. After that, an angle is calculated at each point with respect to the first SVD direction, yielding a histogram that depicts the intensity of each stain. At this point, all of the intensity histograms are scaled to the same pseudo-maximum and compared to each other. Lastly, the concentration of each stain is determined by using the H &E matrix of the OD values and stain normalization is performed. Ultimately, using the H &E matrix with the normalized stain concentration, a normalized image is created. Recently, Vahadane et al.^35^ developed a stain separation framework, called structure-preserving colour normalization (SPCN), which aimed to preserve the structure information of the source image. First, an RGB image is converted to OD values using Lambert-Beer’s law. Then, for stain separation, a sparseness constraint ($\lambda$) is added to the optimization problem to reduce the solution space of the non-negative matrix factorization (NMF), called Sparse NMF (SNMF). In other words, a sparse constraint ($\lambda$) is added to the NMF to effectively separate the stains. Next, the proposed SNMF is used to estimate the color appearances and stain density maps of source and target images. Finally, a normalized image is generated by combining the scaled density map of a source image with the color appearance of a target image. Further theoretical and mathematical details of the aforementioned normalization techniques can be found in their respective original works^32–35^ as well as in the review paper^37^. For the implementation, we utilized Warwick’s Stain Normalization Toolbox (https://github.com/TissueImageAnalytics/tiatoolbox). Figure 2 depicts an example of a source image, a target image, and four normalized images using the above-mentioned practices.

### Training procedure

**Figure 3:**
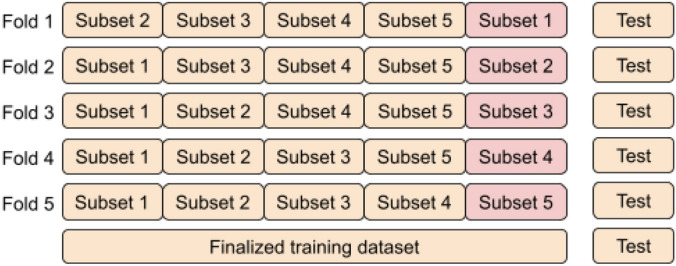
An illustration of the training process based on 5-fold cross-validation.

We selected 80 percent of the images for training and the remaining 20 percent for testing, with an equal percentage of images from each of the four classes. Next, following^8,38^ we applied 5-fold cross-validation on the training dataset, which means that the training dataset (80%) is split into five equal subsets. Among these, four parts (64%) were used for training and one part (16%) was used for validating (evaluating) the model. After finalizing the model, we included the validation part into the training dataset and retrained the model with all 80% of the images. Of note, the test subset is always the same for all the models. All these details are given in Table 2 and illustrated in Fig. 3.

**Table 2:** Selection criteria for training, validation, and test images.

	Colsanitas dataset				Extended colsanitas dataset				Percentage (%)
	Nor.	Ben.	Ins.	Inv.	Nor.	Ben.	Ins.	Inv.	
Train	384	160	160	736	384	640	640	736	64
Valid	96	40	40	184	96	160	160	184	16
Test	120	50	50	230	120	50	50	230	20
Total	600	250	250	1150	600	850	850	1150	100

**Table 3:** Parameters and their values used in in-place data augmentation.

Parameters of ImageDataGenerator	Selected values
Zoom range	0.2
Rotation range	0.2
Width shift range	0.2
Height shift range	0.2
Horizontal flip	False
Vertical flip	False
Fill mode	Reflect

### In-place data augmentation

In-place data augmentation or on-the-fly data augmentation is a technique in which a batch of original images is transformed into its new variation during each and every epoch of the training process. By employing this approach, we want to ensure that the model experiences new variations of input images at each epoch during the training process. To achieve this, we employed ImageDataGenerator provided by Tensorflow deep learning library^39^. The whole process of in-place data augmentation is as follows: (1) First, ImageDataGenerator takes a batch of input images. (2) Then, it transforms every image in the input batch by applying a series of random translations and rotations. In this work, we set “rotation range = 0.2” which corresponds to a random rotation between [− 20, 20] degrees. However, it usually rotates some pixels out of the image frame, leaving empty pixels within the image, which we filled using “fill mode = reflect mode”. Similarly, we specified “ width and height shift range = 0.2” which indicates the percentage of width or height of the image to be shifted randomly, either towards left/right for width or up/down for the height. Also, we selected “zoom range = 0.2” which specifies random zoom-in operation. However, we did not apply horizontal or vertical shifts operation because we already did these shifts when expanding the Colsanitas dataset. (3) Finally, it returns the randomly transformed batch of images. All the parameters and their selected values are provided in Table 3.

**Figure 4:**
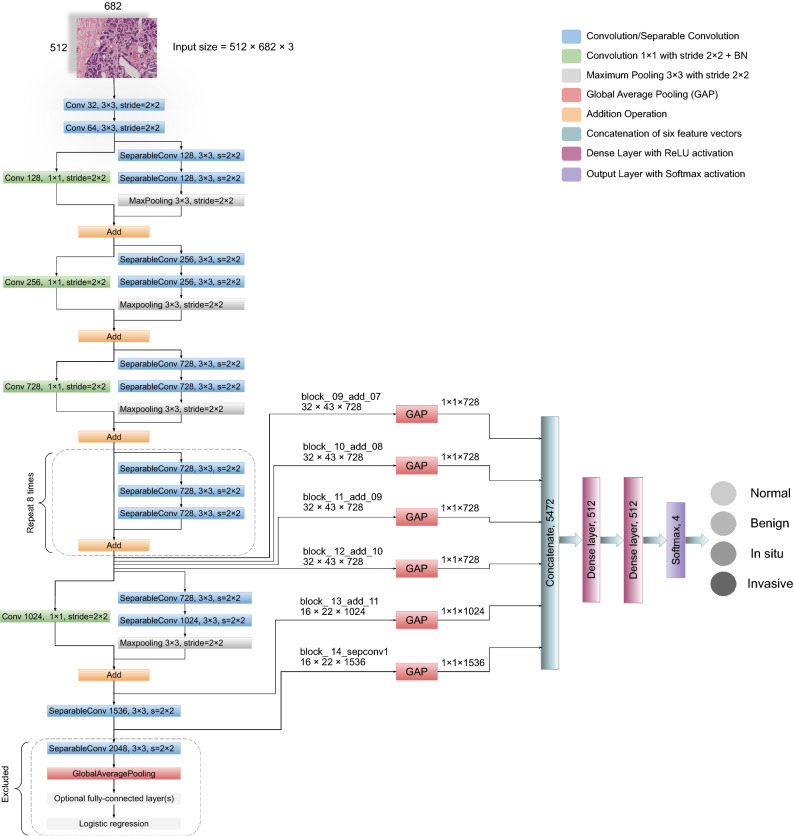
The complete framework of our proposed model is illustrated along with all the layers. For every input image, six different features are extracted followed by the global average pooling. These multilevel features are then concatenated (merged) horizontally to form a single vector of $1\times 1\times 5472$ which is used for classification.

### Proposed model

A straightforward way to increase the performance of a neural network is to increase the number of layers (length) and the number of units at each layer (width). However, the downsides of uniformly increasing network size include a larger number of parameters and computational resources^19^. Therefore, to address the issues of computational efficiency and the number of parameters, Szegedy et al.^19^ introduced the concept of Inception in 2015. The inception module leverages the idea of “network-in-network”^40^ for dimensionality reduction. Also, it convolves an input with different sized filters and concatenates the output. Specifically, the Inception-v1 or GoogleNet, based on inception modules, utilized 12 times fewer parameters than AlexNet^17^ and won the ILSVRC in 2014. In the following years, Inception-v2 or Batch Normalization^41^, Inception-v3^42^, and Inception-v4^43^ were introduced, which are considered to be the improved versions of Inception-v1^19^. In addition to the Inception-v4 architecture, the Inception-ResNet-v1 and Inception-ResNet-v2 models were introduced, which utilized residual connections together with Inception modules^43^. Leveraging inception modules in conjunction with residual connections led to the development of an efficient architecture, called Xception network, which stands for “Extreme Inception”^25^. The Xception is an efficient network which mainly depends on two crucial things: 1) depthwise separable convolution and 2) shortcuts between convolution blocks as in ResNet architecture^20^. Overall, the Xception model has 36 convolutional layers structured together into 14 modules, with each module having a linear residual connection around it, except the first and last one, as shown in Fig. 4.

Our proposed model leveraged the Xception network^25^ to retrieve robust and abstract features from the intermediate layers, as shown in Fig. 4. First, the model takes an RGB image of height 512 and width 682 at its input layer. We reduced the dimension of original images in such a way that the ratio of height and width remained the same. In this way, we preserve the original structure of images, unlike Kassani et al.^29^ that used the dimension of $512 \times 512$. Then, following^25,27,29^, we utilized global average pooling (GAP) on six different layers to obtain the corresponding feature vectors. GAP layers help to decrease the number of parameters and to reduce the overfitting^26^. It is worth mentioning that before finalizing these six layers, we checked the results of different layers from the last seven blocks of the Xception network on the original dataset using k-fold cross-validation. We found that these six layers offered consistent performance in classifying each class with minimal variation. After that, we concatenated (merged) these vectors horizontally to acquire the finalized vector of the dimension 5472 pixels for each image. After the images are converted to their corresponding feature vectors, we trained two dense layers of 512 nodes with Rectified Linear Unit (ReLU) activation function. Lastly, the output layer is comprised of four nodes with Softmax activation and is used for the classification of the given images into four categories. The Softmax function transforms a vector *k* real-valued numbers into a vector of *k* probabilities that sum to 1, as explained in^15^. In our case, the input to the Softmax function is a real-valued vector with $k=4$, whereas its output is a vector of $k=4$ probabilities that sum to 1. The mathematical explanation of softmax function is given in equation 1 and is described in^15^.

1 $$Softmax({\varvec{{z}}})_i = \frac{exp(z_i)}{\sum _{j=1}^{k} exp(z_j)}$$

Where ${\varvec{{z}}}=(z_1, z_2, z_3, z_4)$ is the input vector to the Softmax function and *k* is the number of classes. Moreover, $exp(z_i)$ shows the exponential of the $i^{th}$ real-valued number in the input vector and its value is always positive. Laslty, the normalization term $\sum _{j=1}^{k} exp(z_j)$ depicts the sum of exponential of all the input real-valued numbers and its value is also always positive. In this way, we get a vector of probabilities that sums to 1.

### Implementation setup

We implemented all the experiments using Python version 3.8.5 and TensorFlow 2.4.1^39^, installed on a standard computer machine with two Nvidia GeForce GTX 2070 graphical processing units (GPUs) support. Furthermore, the machine has a RAM of 32.0 GB and holds a 3.60 GHz Intel® Core™ i9-9900K processor with 16 logical threads and 16 MB of cache memory. We followed the distributed training approach of TensorFlow^39^ by using both the GPUs using “tf.distribute.MirroredStrategy(devices=[’/gpu:0’,’/gpu:1’])” strategy.

### Model evaluation

The classification performance of the proposed framework leverages the elements of confusion matrix, also known as contingency table^8,44^. For multiclass classification problem, we defined the elements of the confusion matrix in terms of the target class and non-target class, which can be applied to every individual class^44^. For instance, the target class could be invasive and non-target class could be non-invasive. True Positive (TP) refers to the images that are correctly classified as the target class (invasive), and False Positive (FP) shows the non-target images (non-invasives) that are falsely classified as the target class (invasive). Whereas, False Negative (FN) indicates the images of target class (invasive) classified as non-target class (non-invasive), and True Negative (TN) denotes the correctly classified non-target images (non-invasive). Of note, FP is also called *type I error* and FN is also called *type II error* in the literature. Furthermore, following^45^, we assessed the performance of our proposed model using receiver operating characteristic (ROC) curves and precision-recall (PR) curves along with their area under the curve (AUC) values for every class (one-vs-rest method) for the original and normalized datasets. Lastly, we computed the Cohen’s kappa statistic for the original as well as normalized datasets.

- Precision: It calculates the exactness of a model and defines the ratio of images correctly classified as the target class (invasive) out of all predicted same-class images.
  2 $${\text {Precision}} = \frac{TP}{TP + FP}$$
- Sensitivity: Sensitivity, also known as recall, evaluates the completeness of a model. It determines the ratio of images accurately classified as the target class (invasive) out of all actual same-class images.
  3 $${{Sensitivity}} = \frac{TP}{TP + FN}$$
- Accuracy: It computes the correctness of a model and is defined as the proportion of the number of accurately classified images out of total actual test images.
  4 $${\text {Accuracy}} = \frac{TP+FN}{TP + TN + FP+ FN}$$
- F1-score: It indicates the harmonic average of precision and recall and is commonly employed to optimize a model for either precision or recall.
  5 $${\text {F1-score}} = \frac{2*Precision*Recall}{Precision + Recall}$$
- ROC Curve: The ROC curve shows a relationship between true positive rate (TPR) and false positive rate (FPR) at different thresholds. TPR is also called sensitivity or recall, whereas FPR is equivalent to 1-specificity. An ROC curve depicts that increasing TPR results in also increasing FPR and vice versa. The mathematical formula of TPR is shown in equation 3 whereas that of FPR is provided in equation 6.
  6 $${\text {FPR}} = \frac{FP}{FP+TN}$$
- PR Curve: The PR curve shows an inverse relationship between precision and recall at different thresholds. A PR curve illustrates that increasing precision value results in decreasing recall score and vice versa. The mathematical formula of precision is given in equation 2 whereas that of recall is given in equation 3.
- Cohen’s kappa: It calculates the degree of agreement between the true values and predicted values. It is widely used in to handle multiclass and imbalanced dataset problems. Its mathematical formula is provided in equation 7 where $p_o$ and $p_e$ represent observed and expected agreements, respectively.
  7 $${\text {k}} = \frac{p_o - p_e}{1 - p_e}$$

### Hyperparameter optimization

Neural networks can learn complicated patterns between their inputs and outputs automatically^14,15^. However, many of these input-output connections, may be the result of sampling noise that prevailed during training, but may not exist in the test dataset. This can result in an overfitting problem and thus reduce the prediction ability of a deep learning model. To that end, it is crucial to follow the process of hyperparameter tuning to obtain the generalized predictive performance of the proposed network. In this paper, we followed the 5-fold cross-validation approach (see “Training procedure” section) to get the best set of hyperparameters. The procedure followed to obtain the optimum hyperparameters values is as follows: For our multiclass classification task, we first selected categorical cross-entropy as an objective function. Then, we employed Adam (adaptive moment estimation) algorithm^8,46^ during the training to optimize the model through 1000 epochs. At this point, we checked three variants of learning rates (0.001, 0.0001, 0.00001) and three distinct batch sizes (16, 32, 64) based on recently published studies^8,29^. We found that the learning rate of 0.00001 together with a batch size of 64 worked well in reducing the generalization gap between training and validation loss. Next, we saved the weights of five models which resulted from the lowest validation loss, and evaluated the predictive performance of each model on the unseen test dataset. Importantly, we aimed to maximize the mean value of test accuracy while minimizing the standard deviation after checking the predictive abilities of five individual models. For the final model, we trained the proposed framework with all the training images (training and validation) and saved the weights of the optimum model based on the minimum validation loss. Lastly, we employed these weights to predict the classes of the test images. Importantly, we used the default parameters specified in the original architecture of the Xception paper for the convolutional filters, pooling filters, strides, and padding^25^. All the hyperparameters and their optimal values used in this study are presented in Table 4.

**Table 4:** The optimal hyperparameters of our proposed model.

Hyperparameters	Optimal values
Train approach	5-fold cross-validation
Loss function	Categorical cross-entropy
Optimizer	Adam
Learning rate	0.00001
Batch size	64
Convolution	$1\times 1, 3\times 3, 5\times 5$
Maxpooling	$2\times 2$ with stride 2
Epochs	1000
Dropout	0.1
Regularizer	*L*2

## Results

In this section, we explained and compared the classification performance of our proposed framework by considering the original (unnormalized) and normalized images.

### Results without normalization

**Figure 5:**
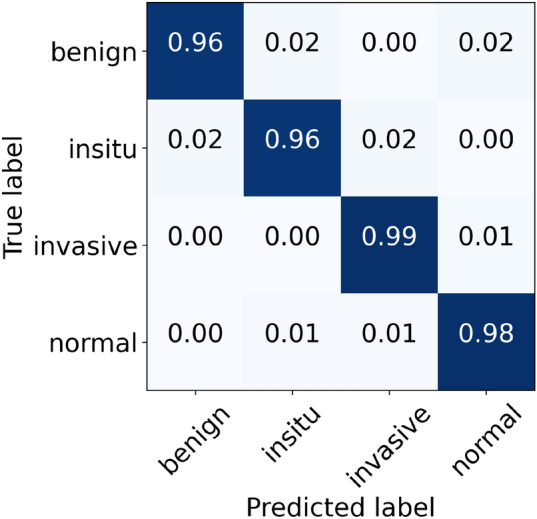
The final normalized confusion matrix of original dataset.

**Figure 6:**
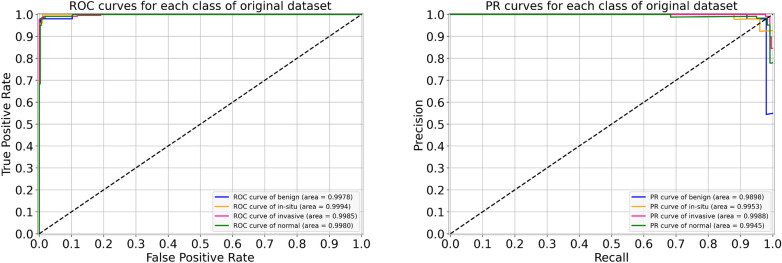
For the original dataset, the left side shows ROC curves for each class with an average AUC-ROC of 0.998. Whereas the right side depicts its PR curves for every class with a mean AUC-PR of 0.995.

For the original (unnormalized) dataset, the performance metrics of our proposed model are provided in Table 5. During the cross-validation, we reported the highest accuracy of 96.88% during folds 1, 2, and 4, whereas the lowest accuracy of 95.33% during fold 5, which led to a mean accuracy of 96.22% $(\pm 0.66)$. The finalized model offered an accuracy value of 98.00%, as shown in Table 5. Specifically, for in situ and invasive carcinomas, we reported sensitivity values of 96.00% and 99.00%, respectively. Similarly, for benign lesions, we found a sensitivity score of 96.00% which is similar to that of in situ carcinoma. The finalized results of all the four classes using the original dataset are shown in Fig. 5. Furthermore, the ROC and PR curves for every class of the original dataset along with their AUC scores are depicted in Fig. 6. The AUC-ROC values vary from 0.998 to 0.999 whereas the AUC-PR values range from 0.990 to 0.999, as displayed in Fig. 6. Of note, the accuracy and loss curves of the original dataset are provided with every normalized dataset for a better visualization and comparison, and are discussed within the next subsections.

**Table 5:** Evaluation metrics of our proposed model using the original dataset.

Folds	Confusion matrices					Performance evaluation					
	Predict $\rightarrow$ Actual $\downarrow$	Ben.	Ins.	Inv.	Nor.	Prec.	Rec.	F1	Test	Accuracy (%)	Kappa
Fold 1	Benign	43	3	2	2	1.00	0.86	0.92	50	96.88	0.951
	In situ	0	49	1	0	0.91	0.98	0.94	50		
	Invasive	0	1	226	3	0.98	0.98	0.98	230		
	Normal	0	1	1	118	0.96	0.98	0.97	120		
Fold 2	Benign	48	1	1	0	0.96	0.96	0.96	50	96.88	0.952
	In situ	2	48	0	0	0.89	0.96	0.92	50		
	Invasive	0	4	222	4	0.99	0.97	0.98	230		
	Normal	0	1	1	118	0.97	0.98	0.98	120		
Fold 3	Benign	47	0	2	1	0.98	0.94	0.96	50	96.00	0.937
	In situ	1	48	1	0	0.96	0.96	0.96	50		
	Invasive	0	1	226	3	0.95	0.98	0.97	230		
	Normal	0	1	8	111	0.97	0.93	0.94	120		
Fold 4	Benign	47	1	0	2	0.94	0.94	0.94	50	96.88	0.952
	In situ	2	47	1	0	0.94	0.94	0.94	50		
	Invasive	0	1	226	3	0.99	0.98	0.98	230		
	Normal	1	1	2	116	0.96	0.97	0.96	120		
Fold 5	Benign	46	2	1	1	0.85	0.92	0.88	50	95.33	0.928
	In situ	3	47	0	0	0.90	0.94	0.92	50		
	Invasive	0	2	224	4	0.99	0.97	0.98	230		
	Normal	5	1	2	112	0.96	0.93	0.95	120		
Final	Benign	48	1	0	1	0.98	0.96	0.97	50	98.00	0.969
	In situ	1	48	1	0	0.96	0.96	0.96	50		
	Invasive	0	0	227	3	0.99	0.99	0.99	230		
	Normal	0	1	1	118	0.97	0.98	0.98	120		

### Results of Reinhard normalization

**Figure 7:**
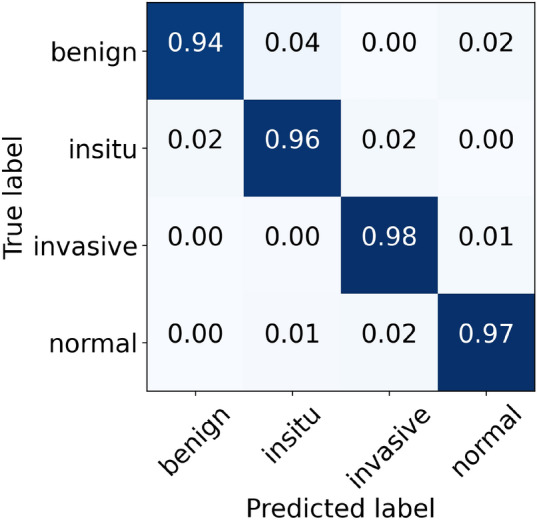
The final normalized confusion matrix of Reinhard dataset.

**Figure 8:**
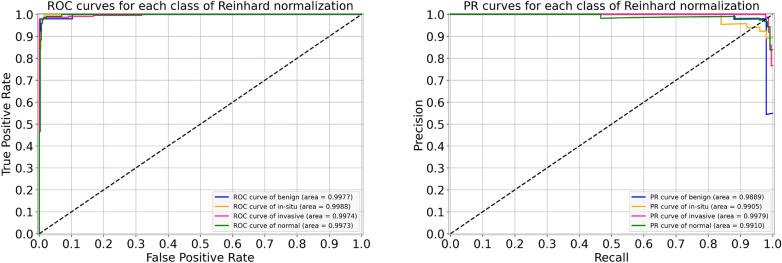
For Reinhard normalization, the left-hand side represents ROC curves for each class with an average AUC-ROC of 0.998. Whereas the right-hand side depicts its PR curves for every class with a mean AUC-PR of 0.992.

**Figure 9:**
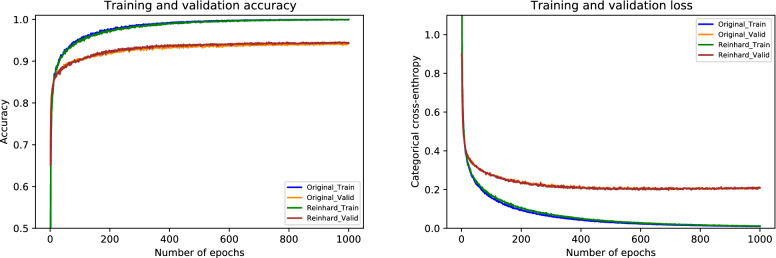
The left-hand side shows a comparison of training and validation accuracy curves of the original dataset and Reinhard normalization. Whereas the right-hand side depicts a comparison of training and validation loss curves of the original dataset and Reinhard normalization.

For the Reinhard normalization, the performance metrics of our proposed architecture are given in Table 6. During the cross-validation, we noted higher accuracy of 97.11% at fold 4 and lower accuracy of 95.33% at fold 5, yielding a mean accuracy of 96.44% $(\pm 0.68)$. The finalized model attained an accuracy of 97.33%, as stated in Table 6. Especially for in situ carcinoma, we observed a sensitivity of 96.00% which is equivalent to that of the original dataset. Whereas for invasive carcinoma, we noted a sensitivity of 98.00% which is 1.00% lower than the original dataset. These finalized results of all the four classes using the Reinhard-based normalized dataset are portrayed in Fig. 7. In addition, the ROC and PR curves for each class of the Reinhard normalization together with their AUC values are illustrated in Fig. 8. In this case, the AUC-ROC values range from 0.997 to 0.999 whereas AUC-PR scores vary from 0.989 to 0.998, as shown in Fig. 8. The accuracy curves of Reinhard normalization along with the original ones are shown on the left side of Fig. 9, whereas their corresponding loss curves are depicted on the right side of Fig. 9. It can be seen that there is no significant difference in these curves. Based on these results, we concluded that although the Reinhard normalization achieved a competitive classification performance, it could not outperform results of the original (unnormalized) dataset.

**Table 6:** Evaluation metrics of our proposed model using Reinhard normalization

Folds	Confusion matrices					Performance evaluation					
	Predict $\rightarrow$ Actual $\downarrow$	Ben.	Ins.	Inv.	Nor.	Prec.	Rec.	F1	Test	Accuracy (%)	Kappa
Fold 1	Benign	43	4	1	2	1.00	0.86	0.92	50	96.44	0.945
	In situ	0	49	1	0	0.89	0.98	0.93	50		
	Invasive	0	1	225	4	0.98	0.98	0.98	230		
	Normal	0	1	2	117	0.95	0.97	0.96	120		
Fold 2	Benign	46	2	0	2	0.98	0.92	0.95	50	96.88	0.952
	In situ	1	49	0	0	0.89	0.98	0.93	50		
	Invasive	0	3	223	4	1.00	0.97	0.98	230		
	Normal	0	1	1	118	0.95	0.98	0.97	120		
Fold 3	Benign	47	2	1	0	0.98	0.94	0.96	50	96.44	0.944
	In situ	1	48	1	0	0.92	0.96	0.94	50		
	Invasive	0	1	226	3	0.97	0.98	0.97	230		
	Normal	0	1	6	113	0.97	0.94	0.96	120		
Fold 4	Benign	47	2	0	1	0.96	0.94	0.95	50	97.11	0.955
	In situ	1	47	1	1	0.94	0.94	0.94	50		
	Invasive	0	0	227	3	0.99	0.99	0.99	230		
	Normal	1	1	2	116	0.96	0.97	0.96	120		
Fold 5	Benign	47	3	0	0	0.87	0.94	0.90	50	95.33	0.928
	In situ	2	47	0	1	0.87	0.94	0.90	50		
	Invasive	2	2	223	3	0.99	0.97	0.98	230		
	Normal	3	2	3	112	0.97	0.93	0.95	120		
Final	Benign	47	2	0	1	0.98	0.94	0.96	50	97.33	0.959
	In situ	1	48	1	0	0.92	0.96	0.94	50		
	Invasive	0	1	226	3	0.99	0.98	0.98	230		
	Normal	0	1	2	117	0.97	0.97	0.97	120		

### Results of Ruifrok normalization

**Figure 10:**
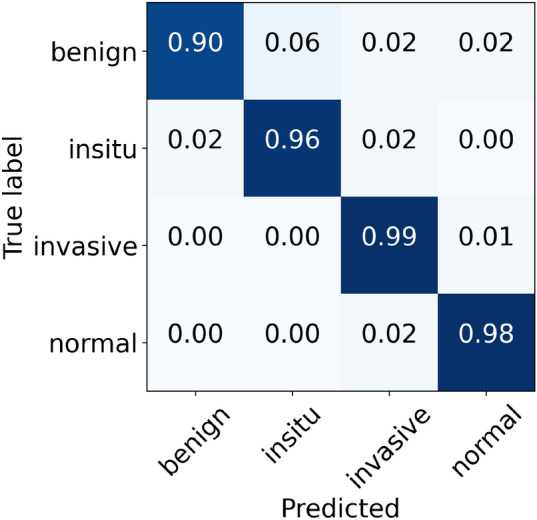
The final normalized confusion matrix of Ruifrok dataset.

**Figure 11:**
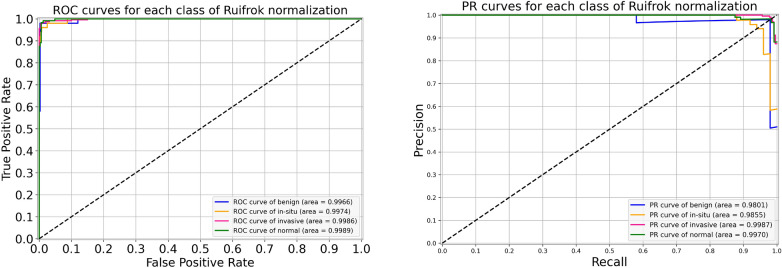
For Ruifrok normalization, the left side represents ROC curves for an individual class with an average AUC-ROC of 0.998. Whereas the right side depicts its PR curves for every class with a mean AUC-PR of 0.990.

**Figure 12:**
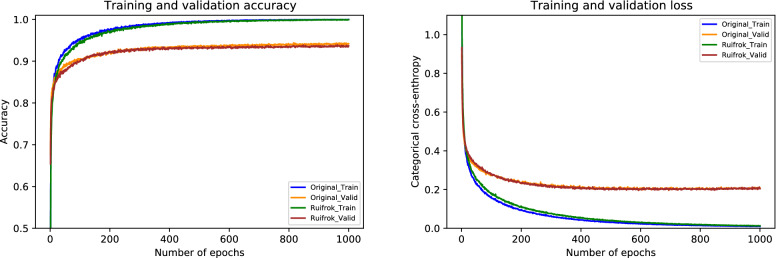
The left side demonstrates a comparison of training and validation accuracy curves of the original dataset and Ruifrok normalization. Whereas the right side illustrates a comparison of training and validation loss curves of the original dataset and Ruifrok normalization.

For the Ruifrok normalization, the performance metrics of our proposed framework are presented in Table 7. During the cross-validation, we observed a highest accuracy of 96.88% during fold 2 and a lowest accuracy of 96.00% during fold 5, which resulted in a mean accuracy of 96.31% $(\pm 0.37)$. The finalized model yielded an accuracy of 97.33%, as mentioned in Table 7. Particularly, the sensitivity for in situ class is 96.00%, which is equal to both the original and the Reinhard normalization. Likewise, the sensitivity for invasive class is 99.00%, which is the same as that of the original but 1.00% higher than the Reinhard normalization. These optimal results of all the classes for the Ruifrok-based normalized dataset are depicted in Fig. 10. Moreover, the ROC and PR curves for an individual class of the Ruifrok normalization in conjunction with their AUC scores are provided in Fig. 11. In this case, the AUC-ROC values range from 0.997 to 0.999 whereas the AUC-PR scores range from 0.980 to 0.999, as demonstrated in Fig. 11. The comparison of accuracy curves, in this case, is shown on the left block of Fig. 12, whereas their corresponding loss curves are illustrated on the right block of Fig. 12. Like the Reinhard normalization, it can be seen that there is no significant difference in these curves. Thus, it can be concluded that the classification performance using the Ruifrok normalization is the same as Reinhard normalization in terms of accuracy.

**Table 7:** Evaluation metrics of our proposed model using Ruifrok normalization

**Folds**	Confusion matrices					Performance evaluation					
	Predict $\rightarrow$ Actual $\downarrow$	Ben.	Ins.	Inv.	Nor.	Prec.	Rec.	F1	Test	Accuracy (%)	Kappa
Fold 1	Benign	42	6	0	2	0.98	0.84	0.90	50	96.22	0.941
	In situ	1	48	1	0	0.84	0.96	0.90	50		
	Invasive	0	2	225	3	0.99	0.98	0.98	230		
	Normal	0	1	1	118	0.96	0.98	0.97	120		
Fold 2	Benign	48	2	0	0	0.98	0.96	0.97	50	96.88	0.952
	In situ	1	48	1	0	0.89	0.96	0.92	50		
	Invasive	0	3	222	5	0.99	0.97	0.98	230		
	Normal	0	1	1	118	0.96	0.98	0.97	120		
Fold 3	Benign	44	3	0	3	0.96	0.88	0.92	50	96.00	0.937
	In situ	2	45	3	0	0.94	0.90	0.92	50		
	Invasive	0	0	226	4	0.97	0.98	0.98	230		
	Normal	0	0	3	117	0.94	0.97	0.96	120		
Fold 4	Benign	46	3	0	1	0.94	0.92	0.93	50	96.44	0.945
	In situ	1	47	2	0	0.94	0.94	0.94	50		
	Invasive	1	0	224	5	0.98	0.97	0.98	230		
	Normal	1	0	2	117	0.95	0.97	0.96	120		
Fold 5	Benign	47	3	0	0	0.90	0.94	0.92	50	96.00	0.938
	In situ	1	47	2	0	0.92	0.94	0.93	50		
	Invasive	1	1	224	4	0.98	0.97	0.98	230		
	Normal	3	0	3	114	0.97	0.95	0.96	120		
Final	Benign	45	3	1	1	0.98	0.90	0.94	50	97.33	0.958
	In situ	1	48	1	0	0.94	0.96	0.95	50		
	Invasive	0	0	227	3	0.98	0.99	0.98	230		
	Normal	0	0	2	118	0.97	0.98	0.98	120		

### Results of Macenko normalization

**Figure 13:**
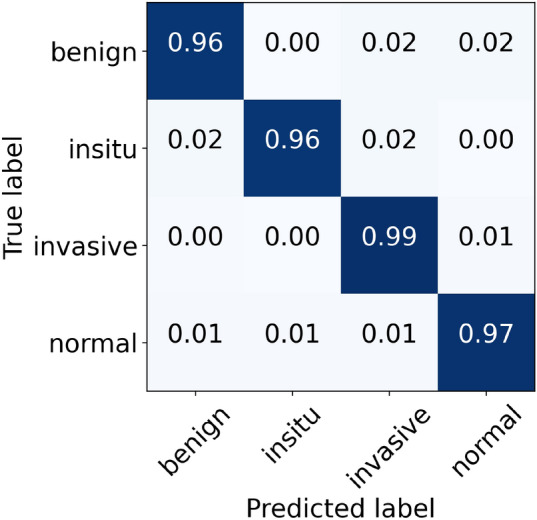
The final normalized confusion matrix of Macenko dataset.

**Figure 14:**
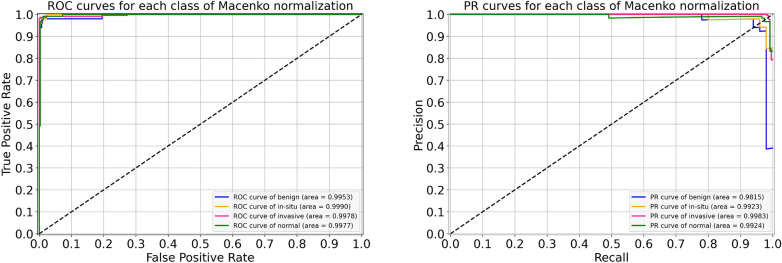
For Macenko normalization, the left block illustrates ROC curves for each class with an average AUC-ROC of 0.997. Whereas the right block depicts its PR curves for the individual class with a mean AUC-PR of 0.991.

**Figure 15:**
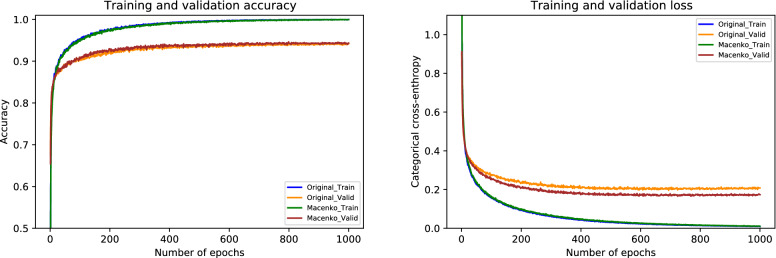
The left graph represents a comparison of training and validation accuracy curves of the original dataset and Macenko normalization. Whereas the right graph portrays a comparison of training and validation loss curves of the original dataset and Macenko normalization.

For the Macenko normalization, the performance metrics of our proposed system are provided in Table 8. During the cross-validation, we observed the uppermost accuracy of 97.11% in fold 4 as well as the lowermost accuracy of 96.00% in fold 3, resulting in a mean accuracy of 96.10% $(\pm 0.88)$. The finalized model got an accuracy of 97.78%, as given in Table 8. In particular, the sensitivity values for in situ and invasive carcinomas, in this case, are 96% and 99%, which are equal to that of the original dataset. These optimal results for all four classes are illustrated in Fig. 13. Besides, the ROC and PR curves for each class of Macenko normalization with their corresponding AUC scores are shown in Fig. 14. Here, AUC-ROC scores vary between 0.995 and 0.999 whereas AUC-PR values range from 0.981 to 0.998, as indicated in Fig. 14. The relationship between accuracy curves is shown on the left portion of Fig. 15, whereas their relative loss curves are depicted on the right portion of Fig. 15. Interestingly, the validation loss improved as compared to the original dataset; however, no considerable changes occurred in validation accuracy. These statistics pointed out that the Macenko-based normalization has slightly outperformed the Reinhard and Ruifrok approaches in terms of accuracy. Also, it offered the same potential as the original dataset in terms of sensitivity for the in situ and invasive carcinomas.

**Table 8:** Evaluation metrics of our proposed model using Macenko normalization

Folds	Confusion matrices					Performance evaluation					
	Predict $\rightarrow$ Actual $\downarrow$	Ben.	Ins.	Inv.	Nor.	Prec.	Rec.	F1	Test	Accuracy (%)	Kappa
Fold 1	Benign	42	3	2	3	0.98	0.84	0.90	50	96.88	0.951
	In situ	0	49	1	0	0.91	0.98	0.94	50		
	Invasive	1	1	227	1	0.98	0.99	0.98	230		
	Normal	0	1	1	118	0.97	0.98	0.98	120		
Fold 2	Benign	46	2	1	1	0.96	0.92	0.94	50	96.44	0.945
	In situ	1	48	0	1	0.91	0.96	0.93	50		
	Invasive	0	2	223	5	0.99	0.97	0.98	230		
	Normal	1	1	1	117	0.94	0.97	0.96	120		
Fold 3	Benign	48	0	0	2	0.96	0.96	0.96	50	96.00	0.937
	In situ	1	48	1	0	0.98	0.96	0.97	50		
	Invasive	0	0	227	3	0.96	0.99	0.97	230		
	Normal	1	1	9	109	0.96	0.91	0.93	120		
Fold 4	Benign	47	1	0	2	0.96	0.94	0.95	50	97.11	0.955
	In situ	1	48	1	0	0.96	0.96	0.96	50		
	Invasive	0	0	227	3	0.98	0.99	0.98	230		
	Normal	1	1	3	115	0.96	0.96	0.96	120		
Fold 5	Benign	48	1	1	0	0.84	0.96	0.90	50	96.22	0.941
	In situ	3	46	1	0	0.96	0.92	0.94	50		
	Invasive	1	0	226	3	0.99	0.98	0.98	230		
	Normal	5	1	1	113	0.97	0.94	0.96	120		
Final	Benign	48	0	1	1	0.94	0.96	0.95	50	97.78	0.965
	In situ	1	48	1	0	0.99	0.96	0.97	50		
	Invasive	1	0	227	2	0.99	0.99	0.99	230		
	Normal	1	1	1	117	0.97	0.97	0.97	120		

### Results of Vahadane normalization

**Figure 16:**
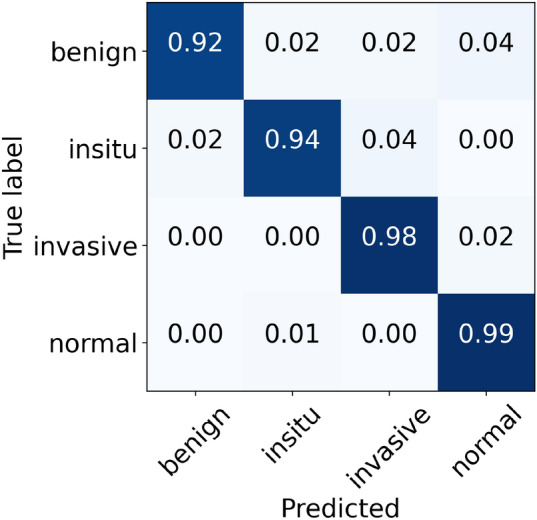
The final normalized confusion matrix of Vahadane dataset.

**Figure 17:**
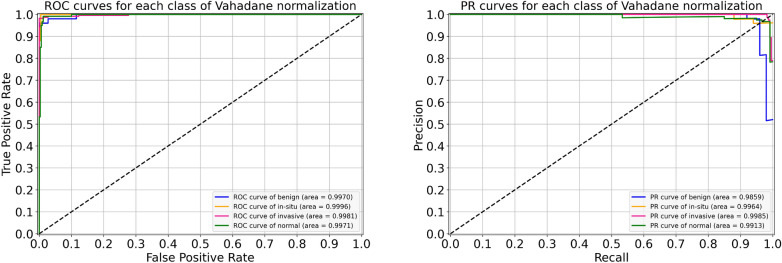
For Vahadane normalization, the left side shows ROC curves for each class with an average AUC-ROC of 0.998. Whereas the right side portrays its PR curves for the individual class with a mean AUC-PR of 0.993.

**Figure 18:**
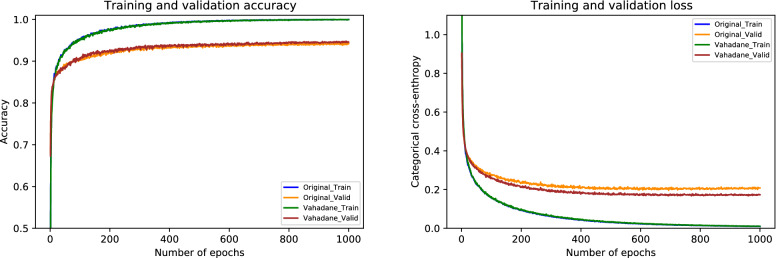
The left side shows a comparison of training and validation accuracy curves of the original dataset and Vahadane normalization. Whereas the right side depicts a comparison of training and validation loss curves of the original dataset and Vahadane normalization.

Lastly, the performance metrics of our suggested model for Vahadane normalization are given in Table 9. During the cross-validation, we found a maximum accuracy of 97.77% during fold 4 and a minimum accuracy of 95.77% during fold 3, with a mean accuracy of 96.57% $(\pm 0.75)$. The accuracy of the finalized model is noted as 97.33%, as indicated in Table 9. Specifically, the sensitivity for in situ carcinoma is 94% which is 2.00% lower than the original dataset. Likewise, the sensitivity for invasive carcinoma is 98% which is 1.00% percent lower than the original dataset. These concluded results of all the four classes are illustrated in Fig. 16. Also, the ROC curves and PR curves for every class of Vahadane normalization along with their AUC values are shown in Fig. 17. In this scenario, the AUC-ROC values vary 0.997 and 0.999 whereas the AUC-PR scores range from 0.986 to 0.996, as mentioned in Fig. 17. The correlation between accuracy curves is shown on the left side of Fig. 18, whereas their corresponding loss curves are displayed on the right side of Fig. 18. Similar to the Macenko normalization, a slight improvement in validation loss can be seen; however, no such improvement occurred in validation accuracy. These statistical analysis show that Vahadane normalization has the same performance as Reinhard and Ruifrok normalization, but is slightly lower than the original and Macenko normalization in terms of accuracy.

**Figure 19:**
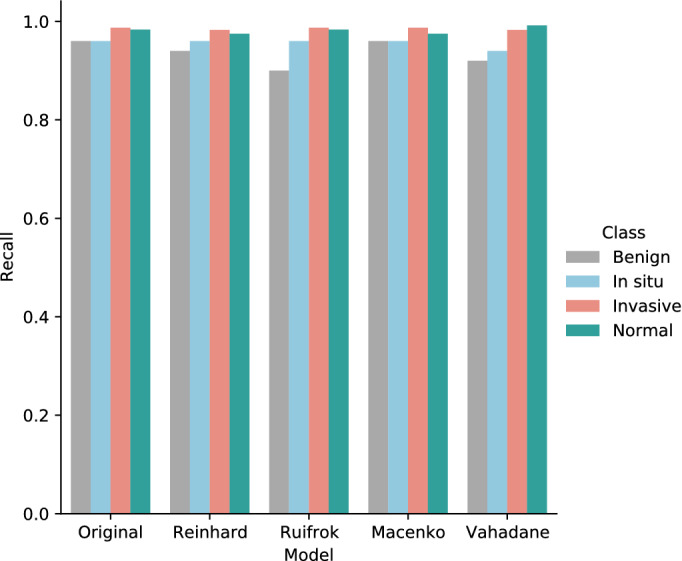
The sensitivity (recall) values of normal, benign, in situ carcinoma, and invasive carcinoma for the original, Reinhard^32^, Ruifrok^33^, Macenko^34^, and Vahadane^35^ datasets.

Finally, the sensitivity values of normal tissue, benign lesion, in situ carcinomas, and invasive carcinomas are collectively illustrated in Fig. 19. Specifically, for in situ carcinomas, the sensitivity of original dataset is equivalent to Reinhard^32^, Ruifrok^33^, and Macenko^34^; however, it is 2% higher than the Vahadane^35^ dataset and this small difference is equivalent to one sample in case of in situ carcinoma. Moreover, for invasive carcinoma, the proposed model offered a higher sensitivity of 99% for original dataset, which is equivalent to Ruifrok^33^ and Macenko^34^ but 1% lower than Reinhard^32^ and Vahadane^35^. In summary, our proposed model achieved generalized performance for the original as well as normalized datasets.

**Table 9:** Evaluation metrics of our proposed model using Vahadane normalization

Folds	Confusion matrices					Performance evaluation					
	Predict $\rightarrow$ Actual $\downarrow$	Ben.	Ins.	Inv.	Nor.	Prec.	Rec.	F1	Test	Accuracy (%)	Kappa
Fold 1	Benign	42	4	2	2	1.00	0.84	0.91	50	96.66	0.948
	In situ	0	49	1	0	0.91	0.98	0.94	50		
	Invasive	0	0	226	4	0.98	0.98	0.98	230		
	Normal	0	1	1	118	0.95	0.98	0.97	120		
Fold 2	Benign	45	2	0	3	0.98	0.90	0.94	50	96.44	0.945
	In situ	1	48	0	1	0.91	0.96	0.93	50		
	Invasive	0	2	222	6	1.00	0.97	0.98	230		
	Normal	0	1	0	119	0.92	0.99	0.96	120		
Fold 3	Benign	46	1	1	2	0.98	0.92	0.95	50	95.77	0.934
	In situ	1	48	1	0	0.96	0.96	0.96	50		
	Invasive	0	0	227	3	0.95	0.99	0.97	230		
	Normal	0	1	9	110	0.96	0.92	0.94	120		
Fold 4	Benign	47	1	0	2	0.98	0.94	0.96	50	97.77	0.965
	In situ	1	48	1	0	0.96	0.96	0.96	50		
	Invasive	0	0	227	3	0.99	0.99	0.99	230		
	Normal	0	1	1	118	0.96	0.98	0.97	120		
Fold 5	Benign	48	1	1	0	0.89	0.96	0.92	50	96.22	0.942
	In situ	2	48	0	0	0.94	0.96	0.95	50		
	Invasive	0	1	223	6	0.99	0.97	0.98	230		
	Normal	4	1	1	114	0.95	0.95	0.95	120		
Final	Benign	46	1	1	2	0.98	0.92	0.95	50	97.33	0.958
	In situ	1	47	2	0	0.96	0.94	0.95	50		
	Invasive	0	0	226	4	0.99	0.98	0.98	230		
	Normal	0	1	0	119	0.95	0.99	0.97	120		

## Discussion

**Table 10:** Comparison of the proposed model based on multilevel features of Xception network with default versions of AlexNet^17^ (baseline), VGG16^18^, VGG19^18^, Inception-v3^42^, and Xception^25^ models as feature extractors.

Model	Dataset	Accuracy (%)	F1-score (%)	Kappa	Training parameters (million)
AlexNet^17^	Original	82.44	77.25	0.720	40.72
	Reinhard	76.66	69.75	0.633	
	Ruifrok	81.55	75.75	0.708	
	Macenko	81.33	75.75	0.702	
	Vahadane	78.89	72.25	0.667	
VGG16^18^	Original	90.44	86.50	0.852	35.95
	Reinhard	88.00	82.50	0.814	
	Ruifrok	87.11	82.50	0.800	
	Macenko	89.55	86.00	0.839	
	Vahadane	89.55	86.25	0.838	
VGG19^18^	Original	87.33	81.75	0.805	41.26
	Reinhard	88.89	82.75	0.824	
	Ruifrok	88.00	81.75	0.814	
	Macenko	89.11	84.25	0.832	
	Vahadane	89.11	83.00	0.832	
Inception-v3^42^	Original	94.66	91.25	0.917	23.08
	Reinhard	94.44	91.25	0.914	
	Ruifrok	94.44	91.50	0.914	
	Macenko	93.55	90.25	0.900	
	Vahadane	93.77	90.00	0.904	
Xception^25^	Original	96.44	95.00	0.945	22.12
	Reinhard	96.66	94.75	0.948	
	Ruifrok	96.66	94.75	0.948	
	Macenko	96.00	94.25	0.938	
	Vahadane	95.56	93.75	0.931	
**Proposed**	Original	98.00	97.50	0.969	20.71
	Reinhard	97.33	96.25	0.959	
	Ruifrok	97.33	96.25	0.958	
	Macenko	97.78	97.00	0.965	
	Vahadane	97.33	96.25	0.958	

The effectiveness of our proposed approach based on multilevel features can be compared with the baseline model (AlexNet^17^) and state-of-the-art deep learning architectures including VGG16^18^, VGG19^18^, Inception-v3^42^, and Xception^25^ networks as feature extractors with their default settings. To that end, we leveraged the same optimal hyperparameters that we selected in our optimized framework, as discussed in the “Hyperparameter optimization” section. Furthermore, we used the same input image size as our proposed model to effectively compare the results, unlike Hao et al.^47^, where the authors selected input image dimensions based on an individual pre-trained CNN model. We trained all of the aforementioned models on 80% of the images, whereas the remaining 20% of the images were used for the test purpose, as explained in the “Training procedure” section. Of note, we chose AlexNet^17^ as a baseline model because it was the first deep CNN model to achieve promising accuracy on the ILSVRC in 2012. Similarly, we considered VGG16^18^ and VGG19^18^ because our recently published study^8^ employed these models to perform binary classification on a dataset that was generated from the same WSI images as used in the current study, as explained in “Colsanitas dataset” section. Furthermore, the reason for selecting the Inception-v3^42^ lies in the simplicity and robustness of its architecture, as discussed in the “In-place data augmentation” section. Finally, the motive behind choosing the plain Xception^25^ is that it could be crucial to evaluate its classification performance along with its modified architecture. Overall, the evaluation metrics of all the models are summarized in Table 10. Further details of these results can be found in the Supplementary Information (SI) file. The detailed comparison of our proposed architecture with each of the aforementioned models is as follows:

The performance metrics of the default AlexNet^17^ model (baseline) as a feature extractor are given in Table 10 (further details can be found in Supplementary Table S1). For the original dataset, it offered an accuracy of 82.44%, F1-score of 77.25%, and Cohen’s kappa score of 0.720. Among the four normalized datasets, it yielded the highest accuracy of 81.55%, F1-measure of 75.75%, and Cohen’s kappa of 0.708 for Ruifrok normalization. However, the baseline model shows overfitting as portrayed in the loss curves of Supplementary Figure S1. Furthermore, it is a computationally expensive model with 40.7 million of training parameters, as mentioned in Table 10. In contrast, our proposed approach leveraged 20.01 million fewer parameters and achieved 15.56 percentage points higher accuracy along with a 24.9 percentage points gain in Cohen’s kappa value for the original dataset.

Similarly, the performance measurements of the default VGG16^18^ model as a feature extractor are also compiled in Table 10 (more details are available in Supplementary Table S2). For the original dataset, it gained an accuracy of 90.44%, F1-score of 86.50%, and Cohen’s kappa statistic of 0.852. It acquired the highest accuracy of 89.55%, F1-measure of 86.25%, and Cohen’s kappa of 0.838 for Vahadane normalization among the four normalized datasets. It can be noticed that VGG16^18^ outperformed the baseline model. Nevertheless, it shows overfitting as illustrated in the loss curves of Supplementary Figure S2. Moreover, like the baseline AlexNet^17^, it is a computationally expensive model with a total number of 35.95 million training parameters, as stated in Table 10. Conversely, our proposed model utilized 15.24 million lower parameters and achieved 7.56 percentage points higher accuracy along with 11.7 percentage points increase in Cohen’s kappa score for the original dataset.

Likewise, the performance metrics of the default VGG19^18^ model as a feature extractor are provided in Table 10 (additional details are given in Supplementary Table S3). It attained an accuracy of 87.33%, F1-measure of 81.75%, and Cohen’s kappa value of 0.805 For the original dataset among the normalized datasets, it reached a maximum accuracy of 89.11%, F1-score of 84.25%, and Cohen’s kappa of 0.832 for Macenko normalization. It can be observed that VGG19^18^ also outperformed the baseline model similar to the VGG16^18^ model. Nonetheless, it exhibits overfitting as portrayed in the loss curves of Supplementary Figure S3. Furthermore, like the baseline AlexNet^17^ and VGG16^18^, it is a computationally expensive model with a total number of 41.26 million training parameters, as stated in Table 10. Contrary to VGG19^18^, our proposed framework utilized 20.55 million fewer parameters and achieved 10.67 percentage points higher accuracy together with 16.4 percentage points increase in Cohen’s kappa score for the original dataset.

Moreover, the performance measurements of the default Inception-v3^42^ model as a feature extractor are also outlined in Table 10 (further details are provided in Supplementary Table S4). For the original dataset, it attained an accuracy of 94.66%, F1-measure of 91.25%, and Cohen’s kappa score of 0.917. Among the normalized datasets, it gained a top accuracy of 94.44%, F1-score of 91.50%, and Cohen’s kappa of 0.914 for Ruifrok normalization. Interestingly, the default Inception-v3^42^ using 23.03 million training parameters offered promising results compared to the baseline AlexNet^17^, and state-of-the-art VGG16^18^ and VGG19^18^ models. However, it shows overfitting as illustrated in the loss curves of Supplementary Figure S4. In contrast, our proposed strategy leveraged 2.37 million lower training parameters and yielded 3.34 percentage points higher accuracy in conjunction with 5.5 percentage points increase in Cohen’s kappa value for the original dataset.

Lastly, the performance metrics of the default Xception^25^ model as a feature extractor are presented in Table 10 (more details can be found in Supplementary Table S5). For the original dataset, it obtained an accuracy of 96.44%, F1-measure of 95.00%, and Cohen’s kappa statistic of 0.945. Among the normalized datasets, it attained the highest accuracy of 96.66%, F1-score of 94.75%, and Cohen’s kappa of 0.948 for both the Reinhard and Ruifrok normalization. It employed 22.21 million of training parameters and outperformed the baseline AlexNetNet^17^ and state-of-the-art VGG16^18^, VGG19^18^, and Inception-v3^42^ models. These results demonstrate that the default Xception model as a feature extractor also offered promising results due to its robust performance in classifying histopathology images^26^. However, the default Xception model started overfitting which can be noticed in the loss curves of Supplementary Figure S5. This can be due to using merely one GAP layer in its default framework. In comparison, our proposed approach used 1.41 million fewer parameters and yielded 1.56 percentage points high accuracy together with a 2.4 percentage points improvement in Cohen’s kappa score for the original dataset.

In summary, these results demonstrate that the baseline AlexNet^17^, as well as the state-of-the-art VGG16^18^ and VGG19^18^, are computationally expensive models. Furthermore, Inception-v3^42^ and Xception^25^ networks offered promising performance but suffered from the overfitting problem. In contrast, our proposed model based on multilevel features of the Xception^25^ network outperformed all the default state-of-the-art frameworks with a fewer number of training parameters. Also, our proposed model offered resistance to overfitting due to the usage of multiple GAP layers^26^. Thus, it can be concluded that when used as a feature extractor, it is better to first check the Xception model with its default setting and then use multiple GAP layers to decrease the overfitting problem^26^. Overall, our proposed model using multilevel features from the intermediate layers of the Xception^25^ network outperformed the baseline as well as state-of-the-art models with their default settings in classifying the breast cancer histopathology images. Interestingly, it provided minimal variance among the results on original and normalized datasets, and thus acted as a generalized deep learning model.

## Conclusion

The purpose of this paper is to leverage deep learning to classify the hematoxylin-eosin-stained breast cancer microscopy images of our collected dataset into normal tissue, benign lesion, in situ carcinoma, and invasive carcinoma. To achieve this, we utilized six intermediate layers of the pre-trained Xception model to extract salient features from input images. We first optimized the proposed architecture on the unnormalized dataset, and then evaluated its performance on normalized datasets resulting from Reinhard, Ruifrok, Macenko, and Vahadane stain normalization procedures. Overall, it is concluded that the proposed approach provides a generalized state-of-the-art classification performance towards the original and normalized datasets. Also, it can be deduced that even though the aforementioned stain normalization methods offered competitive results, they did not outperform the results of the original dataset. In the future, we recommend to use the stain normalization techniques based on generative adversarial networks. Similarly, we suggest exploiting other recently developed pre-trained models by adopting feature extraction and fine-tuning strategies. Furthermore, it would be interesting take to exploit the potential of semi-supervised, unsupervised and self-supervised learning. Lastly, the concepts introduced in this study can be applied to histopathology image classification of different cancers, such as colorectal and lung cancers.

## Supplementary Information

Supplementary Information.

## Data Availability

The data that support the findings of this study are available from the MIFLUDAN project (Elkartek call) by the Basque Country, Spain, but restrictions apply to the availability of these data, which were used under license for the current study, and so are not publicly available. Data are however available from the corresponding author upon reasonable request and with permission of the MIFLUDAN project (Elkartek call) by the Basque Country, Spain.
