# Supplementary material for: Multiclass classification of breast cancer histopathology images using multilevel features of deep convolutional neural network
Source: Sci Rep. 2022 Sep 16;12:15600. doi: 10.1038/s41598-022-19278-2 (PMC9649689; doi:10.1038/s41598-022-19278-2)
Supplement: Supplementary file 1 — Supplementary Information. [file 41598_2022_19278_MOESM1_ESM.pdf]

Multiclass classification of breast cancer histopathology images using multilevel features of deep convolutional neural network

Zabit Hameed[1,*], Begonya Garcia-Zapirain[1], José Javier Aguirre[2,3,4], and Mario Arturo Isaza-Ruget[5]

[1]eVida Research Group, University of Deusto, Bilbao, 48007, Spain
[2]Bioaraba Health Research Institute, Oncology Diagnostics and Therapeutics Area, Department of Pathological Anatomy, University Hospital of Alava. Vitoria, 01009, Spain
[3]NanoBioCel Research Group, School of Pharmacy, University of the Basque Country (UPV/EHU), Vitoria, 01006, Spain
[4]Biokeralty Reseach Institute. Vitoria, 01510, Spain
[5]Fundación Universitaria Sanitas, Bogotá, 110131, Colombia
[*]zabithameed@deusto.es

Table S1. Evaluation metrics of the default AlexNet model (baseline) as a feature extractor using the original and normalized datasets.

	Confusion Matrices					Performance Evaluation					
**Dataset**	Predict → Actual ↓	Ben.	Ins.	Inv.	Nor.	Prec.	Rec.	F1	Test	Accuracy	Kappa
Original	Benign	35	10	3	2	0.73	0.70	0.71	50	82.44%	0.720
	In situ	9	39	2	0	0.70	0.78	0.74	50		
	Invasive	0	5	221	4	0.84	0.96	0.89	230		
	Normal	4	2	38	76	0.93	0.63	0.75	120		
Reinhard	Benign	33	10	3	4	0.52	0.66	0.58	50	76.66%	0.633
	In situ	8	39	2	1	0.64	0.78	0.70	50		
	Invasive	4	9	214	3	0.83	0.93	0.88	230		
	Normal	19	3	39	59	0.88	0.49	0.63	120		
Ruifrok	Benign	31	13	3	3	0.72	0.62	0.67	50	81.55%	0.708
	In situ	9	39	2	0	0.62	0.78	0.69	50		
	Invasive	2	7	215	6	0.85	0.93	0.89	230		
	Normal	1	4	33	82	0.90	0.68	0.78	120		
Macenko	Benign	30	13	4	3	0.75	0.60	0.67	50	81.33%	0.702
	In situ	7	40	3	0	0.63	0.80	0.71	50		
	Invasive	1	6	218	5	0.84	0.95	0.89	230		
	Normal	2	4	36	78	0.91	0.65	0.76	120		
Vahadane	Benign	27	18	2	3	0.77	0.54	0.64	50	78.89%	0.667
	In situ	6	42	2	0	0.53	0.84	0.65	50		
	Invasive	1	8	216	5	0.84	0.94	0.89	230		
	Normal	1	12	37	70	0.90	0.58	0.71	120		

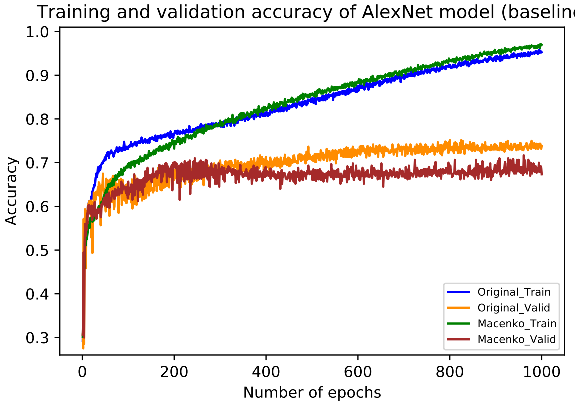

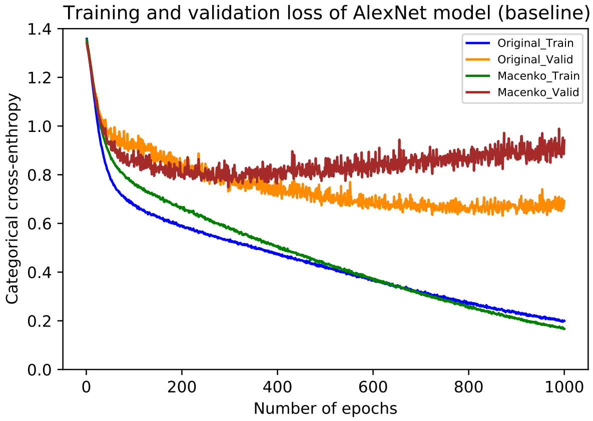

Figure S1. The left-hand side shows training and validation accuracy curves of the original dataset and Macenko normalization on the default settings of the AlexNet model (baseline) as a feature extractor. Whereas the right-hand side depicts training and validation loss curves of the original dataset and Macenko normalization.

Table S2. Evaluation metrics of the default VGG16 model as a feature extractor using the original and normalized datasets.

	**Confusion Matrices**					**Performance Evaluation**					
**Dataset**	Predict → Actual ↓	Ben.	Ins.	Inv.	Nor.	Prec.	Rec.	F1	Test	Accuracy	Kappa
Original	Benign	42	5	1	2	0.79	0.84	0.82	50	90.44%	0.852
	In situ	7	40	3	0	0.77	0.80	0.78	50		
	Invasive	1	4	217	8	0.96	0.94	0.95	230		
	Normal	3	3	6	108	0.92	0.90	0.91	120		
Reinhard	Benign	42	5	1	2	0.67	0.84	0.74	50	88.00%	0.814
	In situ	11	35	4	0	0.78	0.70	0.74	50		
	Invasive	2	3	216	9	0.95	0.94	0.94	230		
	Normal	8	2	7	103	0.90	0.86	0.88	120		
Ruifrok	Benign	38	4	3	5	0.67	0.76	0.71	50	87.11%	0.800
	In situ	8	39	2	1	0.81	0.78	0.80	50		
	Invasive	2	3	215	10	0.94	0.93	0.94	230		
	Normal	9	2	9	100	0.86	0.83	0.85	120		
Macenko	Benign	41	5	1	3	0.76	0.82	0.79	50	89.55%	0.839
	In situ	6	41	2	1	0.82	0.82	0.82	50		
	Invasive	2	3	213	12	0.96	0.93	0.94	230		
	Normal	5	1	6	108	0.87	0.90	0.89	120		
Vahadane	Benign	41	6	1	2	0.82	0.82	0.82	50	89.55%	0.838
	In situ	6	41	3	0	0.77	0.82	0.80	50		
	Invasive	1	3	215	11	0.94	0.93	0.94	230		
	Normal	2	3	9	106	0.89	0.88	0.89	120		

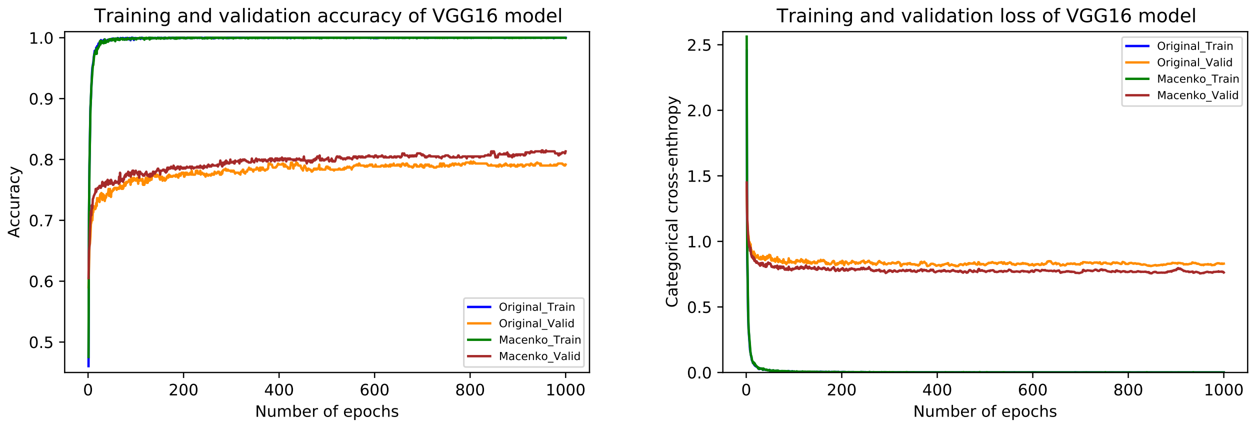

Figure S2. The left-hand side shows training and validation accuracy curves of the original dataset and Macenko normalization on the default settings of the VGG16 model as a feature extractor. Whereas the right-hand side depicts training and validation loss curves of the original dataset and Macenko normalization.

Table S3. Evaluation metrics of the default VGG19 model as a feature extractor using the original and normalized datasets.

	**Confusion Matrices**					**Performance Evaluation**					
**Dataset**	Predict → Actual ↓	Ben.	Ins.	Inv.	Nor.	Prec.	Rec.	F1	Test	Accuracy	Kappa
Original	Benign	45	3	0	2	0.66	0.90	0.76	50	87.33%	0.805
	In situ	15	31	3	1	0.82	0.62	0.70	50		
	Invasive	4	1	210	15	0.96	0.91	0.94	230		
	Normal	4	3	6	107	0.86	0.89	0.87	120		
Reinhard	Benign	46	3	1	0	0.64	0.92	0.75	50	88.89%	0.824
	In situ	16	30	2	2	0.86	0.60	0.71	50		
	Invasive	2	1	218	9	0.96	0.95	0.96	230		
	Normal	8	1	5	106	0.91	0.88	0.89	120		
Ruifrok	Benign	40	7	2	1	0.65	0.80	0.71	50	88.00%	0.814
	In situ	13	34	2	1	0.77	0.68	0.72	50		
	Invasive	4	0	217	9	0.95	0.94	0.95	230		
	Normal	5	3	7	105	0.91	0.88	0.89	120		
Macenko	Benign	44	4	1	1	0.70	0.88	0.78	50	89.11%	0.832
	In situ	12	34	3	1	0.83	0.68	0.75	50		
	Invasive	3	1	213	13	0.96	0.93	0.94	230		
	Normal	4	2	4	110	0.88	0.92	0.90	120		
Vahadane	Benign	43	5	1	1	0.67	0.86	0.75	50	89.11%	0.832
	In situ	13	33	2	2	0.82	0.66	0.73	50		
	Invasive	3	1	216	10	0.96	0.94	0.95	230		
	Normal	5	1	5	109	0.89	0.91	0.89	120		

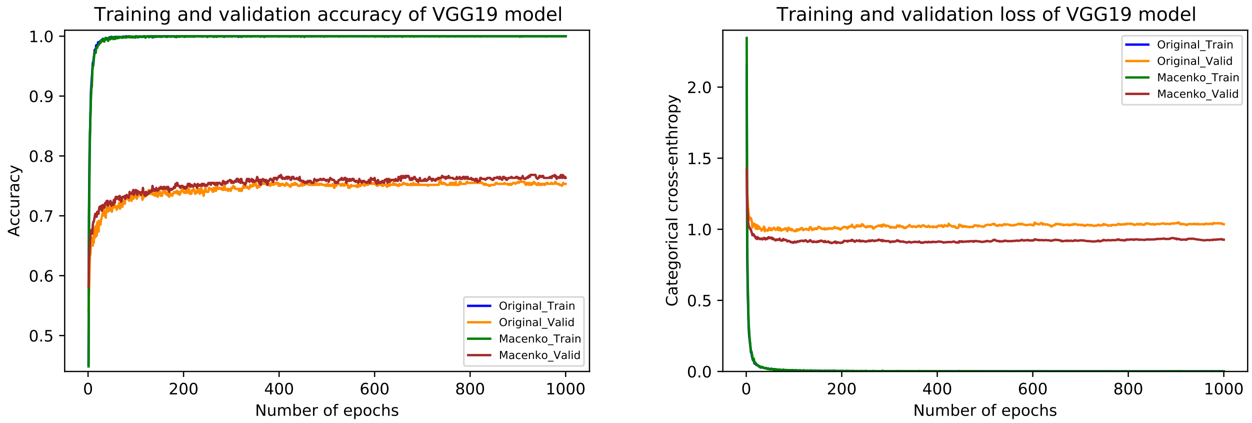

Figure S3. The left-hand side shows training and validation accuracy curves of the original dataset and Macenko normalization on the default settings of the VGG19 model as a feature extractor. Whereas the right-hand side depicts training and validation loss curves of the original dataset and Macenko normalization.

Table S4. Evaluation metrics of the default Inception-v3 model as a feature extractor using the original and normalized datasets.

	**Confusion Matrices**					**Performance Evaluation**					
**Dataset**	Predict → Actual ↓	Ben.	Ins.	Inv.	Nor.	Prec.	Rec.	F1	Test	Accuracy	Kappa
Original	Benign	45	2	0	3	0.82	0.90	0.86	50	94.66%	0.917
	In situ	7	41	2	0	0.91	0.82	0.86	50		
	Invasive	1	1	225	3	0.98	0.98	0.98	230		
	Normal	2	1	2	115	0.95	0.96	0.95	120		
Reinhard	Benign	44	3	0	3	0.83	0.88	0.85	50	94.44%	0.914
	In situ	5	43	0	2	0.90	0.86	0.88	50		
	Invasive	2	1	223	4	0.99	0.97	0.98	230		
	Normal	2	1	2	115	0.93	0.96	0.94	120		
Ruifrok	Benign	44	3	0	3	0.83	0.88	0.85	50	94.44%	0.914
	In situ	5	44	1	0	0.88	0.88	0.88	50		
	Invasive	4	2	218	6	1.00	0.95	0.97	230		
	Normal	0	1	0	119	0.93	0.99	0.96	120		
Macenko	Benign	42	4	0	4	0.84	0.84	0.84	50	93.55%	0.900
	In situ	5	43	1	1	0.88	0.86	0.87	50		
	Invasive	1	1	221	7	0.99	0.96	0.97	230		
	Normal	2	1	2	115	0.91	0.96	0.93	120		
Vahadane	Benign	41	5	1	3	0.82	0.82	0.82	50	93.77%	0.904
	In situ	6	43	0	1	0.83	0.86	0.84	50		
	Invasive	2	3	221	4	0.99	0.96	0.98	230		
	Normal	1	1	1	117	0.94	0.97	0.96	120		

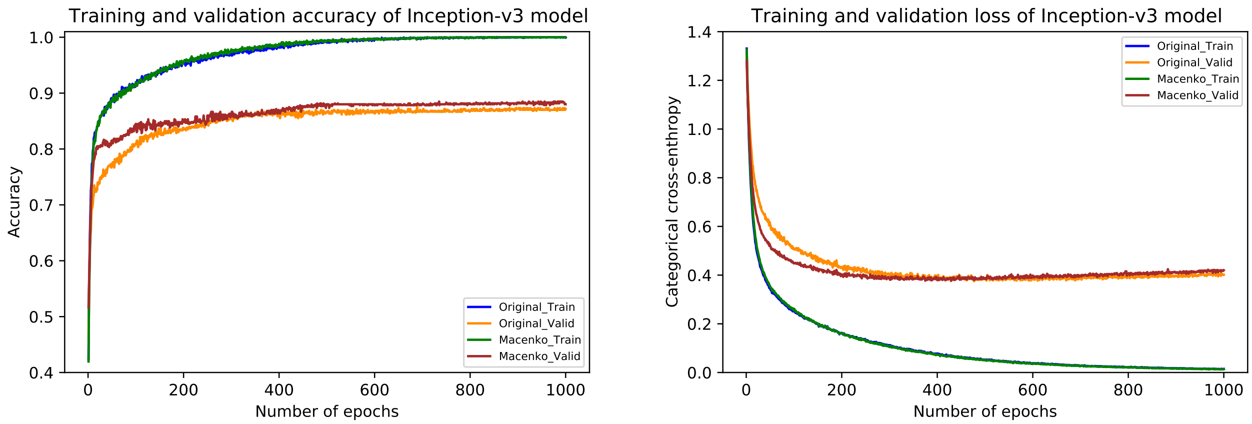

Figure S4. The left side shows training and validation accuracy curves of the original dataset and Macenko normalization on the default settings of the Incepion-v3 model as a feature extractor. Whereas the right side depicts training and validation loss curves of the original dataset and Macenko normalization.

Table S5. Evaluation metrics of the default Xception model as a feature extractor using the original and normalized datasets.

	Confusion Matrices					Performance Evaluation					
**Dataset**	Predict → Actual ↓	Ben.	Ins.	Inv.	Nor.	Prec.	Rec.	F1	Test	Accuracy	Kappa
Original	Benign	47	0	0	3	0.92	0.94	0.93	50	96.44%	0.945
	In situ	3	45	1	1	0.98	0.90	0.94	50		
	Invasive	0	0	225	5	0.99	0.98	0.98	230		
	Normal	1	1	1	117	0.93	0.97	0.95	120		
Reinhard	Benign	47	1	1	1	0.89	0.94	0.91	50	96.66%	0.948
	In situ	5	44	1	0	0.96	0.88	0.92	50		
	Invasive	0	0	227	3	0.99	0.99	0.99	230		
	Normal	1	1	1	117	0.97	0.97	0.97	120		
Ruifrok	Benign	46	2	0	2	0.90	0.92	0.91	50	96.66%	0.948
	In situ	3	47	0	0	0.92	0.94	0.93	50		
	Invasive	0	1	226	3	1.00	0.98	0.99	230		
	Normal	2	1	1	116	0.96	0.97	0.96	120		
Macenko	Benign	47	0	2	1	0.87	0.94	0.90	50	96.00%	0.938
	In situ	4	44	2	0	0.98	0.88	0.93	50		
	Invasive	1	0	225	4	0.98	0.98	0.98	230		
	Normal	2	1	1	116	0.96	0.97	0.96	120		
Vahadane	Benign	46	1	0	3	0.88	0.92	0.90	50	95.56%	0.931
	In situ	4	44	1	1	0.96	0.88	0.92	50		
	Invasive	1	0	225	4	0.98	0.98	0.98	230		
	Normal	1	1	3	115	0.93	0.96	0.95	120		

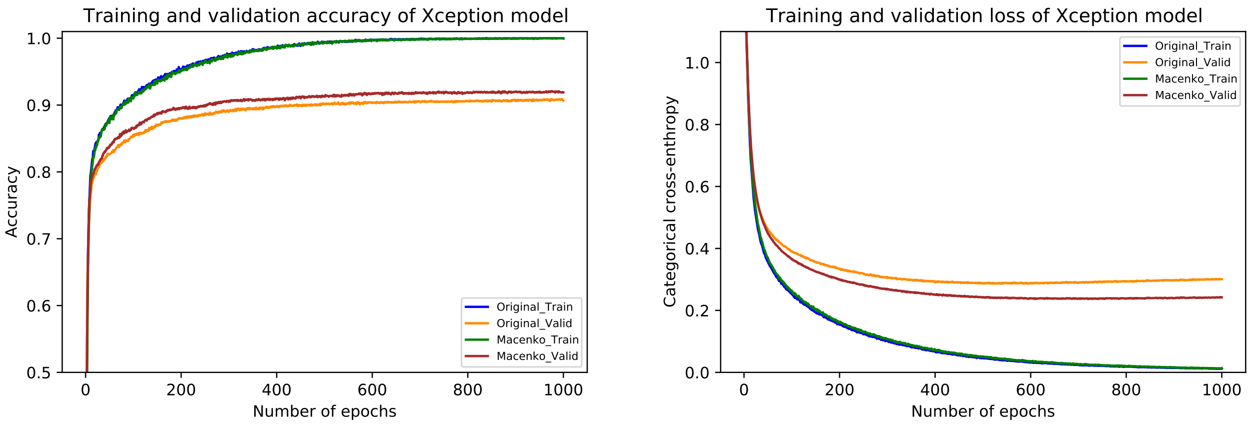

Figure S5. The left side demonstrates training and validation accuracy curves of the original dataset and Macenko normalization on the default settings of the Xception model as a feature extractor. Whereas the right side presents training and validation loss curves of the original dataset and Macenko normalization.
